# Co-Localization of Resistance and Metabolic Quantitative Trait Loci on Carrot Genome Reveals Fungitoxic Terpenes and Related Candidate Genes Associated with the Resistance to *Alternaria dauci*

**DOI:** 10.3390/metabo13010071

**Published:** 2023-01-02

**Authors:** Claude Emmanuel Koutouan, Valérie Le Clerc, Anita Suel, Latifa Hamama, Patricia Claudel, David Halter, Raymonde Baltenweck, Philippe Hugueney, Jean-François Chich, Sitti Anlati Moussa, Clémentine Champlain, Sébastien Huet, Linda Voisine, Sandra Pelletier, Sandrine Balzergue, Wilfried Chevalier, Emmanuel Geoffriau, Mathilde Briard

**Affiliations:** 1 Institut Agro, Université d’Angers, INRAE, IRHS, SFR 4207 QUASAV, F-49000 Angers, France; 2 Université de Strasbourg, INRAE, SVQV UMR-A 1131, F-68000 Colmar, France

**Keywords:** metabolomic, transcriptomic, antifungal activities, *Daucus carota*, leaf blight

## Abstract

Alternaria leaf blight, caused by the fungus *Alternaria dauci*, is the most damaging foliar disease of carrot. Some carrot genotypes exhibit partial resistance to this pathogen and resistance Quantitative Trait Loci (rQTL) have been identified. Co-localization of metabolic QTL and rQTL identified camphene, α-pinene, α-bisabolene, β-cubebene, caryophyllene, germacrene D and α-humulene as terpenes potentially involved in carrot resistance against ALB. By combining genomic and transcriptomic analyses, we identified, under the co-localization regions, terpene-related genes which are differentially expressed between a resistant and a susceptible carrot genotype. These genes include five terpene synthases and twenty transcription factors. In addition, significant mycelial growth inhibition was observed in the presence of α-humulene and caryophyllene.

## 1. Introduction

Plants synthesize a large number of specialized metabolites (about 200,000) involved in many aspects of plant life [1,2]. Terpenoids, with an estimated 20,000 to 40,000 compounds, belong to an important family of these specialized metabolites [3,4]. Monoterpenes and sesquiterpenes are subfamilies of terpenoids and are synthetized through the methylerythritol phosphate (MEP) and the mevalonic acid (MVA) pathways, respectively. Geranyl diphosphate (GPP) and farnesyl diphosphate (FPP) are precursors to mono- and sesquiterpenes, respectively [4]. Monoterpenes and sesquiterpenes are involved in plant flavors, perfumes, thermotolerance, response to light stress, attraction of pollinators or predators of insect pests and defense against microbial pathogens [3,5,6,7]. Their involvement in defense response to fungal pathogens has been shown in different pathosystems [8,9,10]. Terpene synthases (TPS) are key enzymes in terpenoid biosynthesis, giving rise to a large diversity of terpene carbon skeletons. Plant TPS gene families may comprise several dozens of members, which have been classified into eight subfamilies, designated TPS-a to TPS-h, based on sequence and functions. In angiosperms, the TPS-a family contains mostly sesquiterpene and diterpene synthases, while monoterpene synthases belong mostly to the TPS-b and TPS-g clades [11]. In addition to terpene synthases, transcription factors (TF) can regulate gene expression involved in terpene biosynthesis. Since the initial characterization of the R2R3 MYB TF ODORANT1 as a regulator of fragrance biosynthesis in petunia flowers [12], a number of TF involved in the regulation of terpenoid biosynthesis have been characterized in a number of plant species [13].

Alternaria leaf blight is the most damaging foliar disease in carrot, causing burning symptoms on leaves, which make harvesting difficult or impossible in case of a major attack [14]. Symptoms of *A. dauci* appear as brown lesions surrounded or not by a chlorotic halo and then spread to the entire leaf under favorable conditions (average temperature 22–24 °C and high humidity in the range 96 to 100%). The fungus can also contaminate inflorescences, seeds and persist in infected soils [14]. Commercial varieties are only partially resistant and there is still a need for antifungal treatments. The objective of carrot breeders is to develop varieties with higher levels of resistance than those currently available. Deciphering the resistance mechanisms is therefore crucial for this purpose. In previous work, three QTL regions associated with resistance (rQTL) were identified within a segregating population obtained from a cross between a parent susceptible to ALB (S269) and a partially resistant one (R268) [15]. From two other connected populations, PC2 and PC3, obtained from the cross between a susceptible parent H1 and two partially resistant parents I2 and K3, respectively, Le Clerc et al. [16] highlighted new rQTL. Eleven rQTL were identified with four and five most favorable alleles coming from the two resistant parental lines, while two other favorable alleles came from the susceptible one. Global R^2^ were high, i.e., 43% and 52% in 2014 and 2011, respectively. Nevertheless, the mechanisms underlying these rQTL remained unknown. In a previous work, we also showed significant differences in the accumulation of secondary metabolites belonging to the flavonoid and terpenes families in carrot genotypes with different resistance levels to ALB [17].

The objective of the present study is to evaluate the potential role of terpenes in carrot resistance based on a metabolite-QTL (mQTL)-rQTL co-localization approach. For this purpose (i) the terpene contents in plants of the PC3 segregating population were analyzed after *A. dauci* natural infestation in the field; (ii) these terpene contents were used for m-QTL mapping and the co-localization between mQTL and rQTL was analyzed; (iii) a microarray analysis was performed to identify candidate genes underlying co-localizing mQTL–rQTL; and (iv) finally, the antifungal activities of four terpene candidates were tested in vitro.

## 2. Materials and Methods

### 2.1. Plant Materials

A cross between H1, a French susceptible S3 line from the HM Clause company breeding program, and K3, a partially resistant Asiatic S2 line from the Institut Agro breeding program, was realized to develop a F2:3 segregating population named PC3. These two parental lines H1 and K3 are not only very different in terms of susceptibility to *Alternaria dauci*, but also on their metabolite and genetic profiles [16,17].

The development of the PC3 population was described in [16]. Briefly, in order to choose appropriate susceptible and resistant parental lines, different accessions were evaluated in different environments (France, Brazil, field and tunnel) from 1997 to 2006. Based on those trials and parental genetic distance, F1 hybrids were obtained from the cross between H1 and K3 and self-pollinated to provide F2 populations. About 180 individual plants from one of these best segregating F2 populations were self-pollinated to derive the F3 population called PC3 (i.e., 180 F_2:3_ lines). In addition to the parental lines (H1 and K3) and the F_2:3_ lines of PC3, Presto, a susceptible cultivar from Vilmorin, was also used for the trials to ensure pathogen attack. Boléro, another cultivar from Vilmorin, was used as a partially resistant reference.

### 2.2. Experimental Design and Crop Management

For metabolite-QTL detection, two field trials were performed, one in 2015 at Blagon (latitude 44.7835, longitude −0.9319; Gironde, France) and one in 2016 at Ychoux (latitude 44.3333, longitude −0.9667; Les Landes, France). During 2015 and 2016, 137 and 142 F_2:3_ progenies from PC3 with enough seeds available were used, respectively. Two replicates per progeny and ten replicates of parental lines with about 180 seeds over a two linear meter row were sown in each field trial. Presto was sown all along a central row. Two blocks were designed on each side of this Presto row. Replicates of progenies and parental lines were randomly distributed within these two blocks. As described by [17], both trials were done in sandy soils during the optimal carrot growth period. The two fields are located in carrot production areas where *A. dauci* pressure is very high. Each replicate was harvested eight days after natural infestation in both experiments. During both field trials (2015, 2016), pathogen attacks were predicted using the Plant-Plus system^®^ developed by Dacom (http://www.dacom.nl/ (accessed on 7 December 2022)), which considers weather conditions, plant development stage and pathogen concentration in this area of carrot production.

In 2017, the two parental lines (H1 and K3) were sown under tunnel at Angers (latitude 47.4711; longitude −0.5518; Maine et Loire, France) for microarray analysis. Four replicates of each line with 360 seeds were sown over a four linear meter row. The inoculum was prepared from four *Alternaria dauci* strains used by GEVES (Groupe d’Etude et de contrôle des Variétés et des Semences) for resistance testing of carrot varieties before their registration in the French official catalogue (GEVES, personal communication). The four replicates per line were harvested 48 h post inoculation (H48).

### 2.3. Sampling Design

Eight plants per replicate were harvested for mQTL and microarray analyses. The following steps were as described by [17]. Briefly, the plants were cold transported to laboratory, and then two intermediate leaves per plant were bulked from eight plants per replicate, ground in a mortar with liquid nitrogen to obtain fresh powder stored at −80 °C (called below “the roughly ground powder”).

### 2.4. Headspace Solid-Phase Microextraction Followed by Gas Chromatography–Mass Spectrometry (HS-SPME-GC-MS) for Terpene Analyses

SPME vials (20 mL; Macherey–Nagel) containing 25 mg of fresh frozen roughly ground powder were filled with 2 mL of Na_2_SO_3_ solution (10 g/L). 3-octanol (50 µg) was added as an internal standard. Each sample was incubated for 15 min at 35 °C. The volatile compounds were extracted under agitation (250 rpm) with a divinylbenzene/Carboxen/polydimethylsiloxane fiber (1 cm, 23-gauge, 50/30 µm DVB/CAR/PDMS; Supelco, Bellefonte, PA, USA) at 35 °C for 15 min, fitted to a Gerstel MPS2 autosampler. The GC (Agilent 6890 Gas Chromatograph) was fitted with a DB-Wax column (i.d.: 30 m × 0.32 mm; film thickness: 0.5 µm). Helium was used as carrier gas with a column flow rate of 1.3 mL·min^−1^. Volatiles were desorbed from the fiber in the GC inlet (220 °C) for 3 min and separated using the following temperature program: 40 °C for 5 min, increasing by 3 °C/min to 240 °C, and then held for 5 min. The MS (Agilent 5973N Mass Spectrometer) transfer line and ion source temperatures were set at 270 and 230 °C, respectively. The MS was operated in electron ionization mode and positive ions at 70 eV were recorded with a scan range from *m*/*z* 30 to *m*/*z* 300. ChemStation software (G1701DA, Rev D.03.00, Agilent, Santa Clara, CA, USA) was used for instrument control and data processing. The identity of the detected volatiles was determined by comparing their mass spectra with those of authentic standards and spectral libraries. The U.S. National Institute of Standards and Technology (NIST-05a, Gaithersburg, MA, USA), and the Wiley Registry 7th Edition mass spectral libraries were used for identification. Data are presented as normalized peak area per mg of fresh weight.

### 2.5. Correlation between Metabolite Accumulation and Disease Score

For disease evaluation, symptoms on leaves were scored for each replicate with 0–9 severity scale, 0 corresponds to no visible symptoms and 9 to leaves totally blight [18], which means the lower the disease score, the higher the resistance. In 2015, even if disease attack was confirmed by Dacom model and positive *A. dauci* isolation, it was not possible to evaluate symptoms due to too little disease development. Therefore, in order to rely on solid disease evaluation, the results of two previous exactly similar experiments were added to the 2016 results. Thus, three years of scoring were used for the rQTL analysis (2011, 2014 and 2016 in Les Landes), data were autoscaled, i.e., centered to mean, scaled to the standard deviation of the disease score [19] and averaged for each genotype. Terpene contents were expressed as the mean of autoscaled data (2015 and 2016). Spearman correlations were calculated between terpene contents and disease score obtained for each PC3 progeny. Significance of each correlation was estimated with *p*-value calculated from a Student’s t-test. All analyses were performed using RStudio 1.0.136 software.

### 2.6. Resistance and Metabolite-QTL Detection

The genetic map was already described [20] with a few SSR added. The rQTL were already detected for 2011 and 2014 [20]. A new detection was performed to integrate the new markers and to identify the rQTL with the phenotypic evaluation performed in 2016. The QTL detection was performed by regression interval mapping using MCQTL-5.2.6-Linux.sh software [21]. Briefly, the QTL detections were performed on the two bi-parental populations (PC2 and PC3) in a connected way with the additive connected model for disease (rQTL) and with the PC3 population with the additive disconnected model for the detection of metabolite-QTLs. For each trait (disease score and secondary metabolites accumulation), a threshold value for QTL detection and co-factor selection was computed under an *F* test with 1000 permutations. Marker co-factors were selected in a forward method with 90% of the detection threshold value. Then, QTL was detected with the iterative QTL mapping procedure according to the detection threshold value. A QTL was indicated when the LOD (logarithm of odds) exceeded the threshold. A 1 and 1.5 LOD support interval (LOD SI) suitable for the 95% confidence interval were computed for all QTLs. The phenotypic variation (for the disease score) and the accumulation (for the metabolite) explained by each QTL and by all QTLs were calculated and referred as R^2^ and global R^2^, respectively. The mQTL detection was performed by regression interval mapping using MCQTL-5.2.6-Linux.sh software [21]. 

The co-localization zones were obtained using a two-step method: the mQTL and rQTL were mapped and then the genome zones common to their respective 1 LOD SI were defined by the SSR markers flanking these zones.

The mean of the autoscaled values (terpene contents), from the two-year experiments in field (2015 and 2016) were used for mQTL detection. For each trait, broad sense heritability (*H*^2^) were estimated as *H*^2^ = *σG*^2^/*σP*^2^, where *σG*^2^ is the genotypic variance and *σP*^2^ the phenotypic one. The phenotypic variance includes *σP*^2^ = *σG*^2^ *+ σGY*^2^/*Y + σԑ*^2^/*rY*, where *σGY*^2^ is the genotype: environment variance, *Y* is the number of years, *σԑ*^2^ is the residual variance and *r* is the number of replicates.

### 2.7. Transcriptomic Analysis

***RNA extraction*.** Two randomly chosen H48 leaf samples for each of the two H1 and K3 lines harvested in the inoculated part of the 2017 tunnel experiment were used. About 1 g of each roughly ground powder was ground again with liquid nitrogen and iron beads using an MM2 Retsch mixer-mill to obtain very fine powder. RNA was extracted from 50 mg fine powder following the protocol of the NucleoSpin^®^ RNA Plus kit (Macherey–Nagel, Hoerdt, France). RNA was quantified using a NanoDrop ND-1000 (NanoDrop Technologies, Wilmington, DE, USA), and RNA quality was assessed with an Agilent 2100 Bioanalyzer.

***Microarray analysis*.** One hundred nanograms of RNA from each H48 inoculated replicate was amplified and labeled using a Low Input Quick Amp Labeling Kit (Agilent, Les Ulis, France, ref: 5190–2306) as follows. Briefly, mRNA was retro-transcribed into complementary-DNA (cDNA) and a cRNA was synthesized from the cDNA. The cRNA was amplified and labeled with two dyes, cyanine 3 or 5. Then, the cRNA was purified with Rneasy Mini Kit (Qiagen, Courtaboeuf, France, ref: 74106) and purified cRNA was hybridized onto the Agilent–*Daucus carota* v1 chip (Agilent ref: 084550-G4862A) and with Gene Expression Hybridization Kit (Agilent, Les Ulis, France, ref: 5188–5242). After hybridization, washing steps were performed with a Gene Expression Wash Buffer Kit (Agilent, Les Ulis, France, ref: 5188–5327) and the slide was scanned using the InnoScan 710 (Innopsys, Carbone, France) scanner. Data were extracted using the Mapix^®^ (Innopsys, Carbone, France) software.

The *Daucus carota* v1 chip was designed by the BIDefi-IRHS team using the contigs of transcriptome sequencing from [22]. It has been submitted to Gene Expression Omnibus under the accession number GPL25816. The microarray contains three sense and two complementary antisense sequences probes corresponding to 33,978 coding genes. All the probes were designed within the Coding DNA Sequence (CDS) of the corresponding genes and fixed on a 4 × 180 K microarray slide. For the comparison of H1 and K3 inoculated samples, two biological replicates with two technical repetitions per replicate were analyzed in dye-swap, as described by [23].

### 2.8. Statistical Analysis and Highlighting of Differentially Expressed Genes within the Co-Localization Area

Analyses were performed using the R package Limma from Bioconductor. First, data were normalized with the Loess method. Then, the Lmfit function and the eBayes function from the Limma package were used to highlight differentially expressed genes. Background noise (i.e., the average of the lowest intensities plus two times the standard deviation) was subtracted from normalized data [24]. Only transcripts with adjusted *p*-value (Benjamini–Hochberg) <0.01 were considered.

Molecular markers (SSR) flanking mQTL-rQTL co-localization areas were aligned to the carrot genome using the Geneious 10.2.3 software [22,25]. The locus number of each gene inside the co-localization area was extracted and associated with the corresponding differentially expressed gene already identified.

### 2.9. Fungal Growth Inhibition Assays

The *Alternaria dauci* P2 (FRA017) strain with medium aggressiveness [26] was cultivated on V8 agar medium in the dark at 22 °C for 10–15 days. After fungal growth, one infected agar plate was punched out with a 5 mm diameter punch, which was deposited on the side of a 5 cm diameter malt/agar Petri dish. A sterile antibiotic assay paper (Dominique Dutscher, Bernolsheim, France) was deposited at the opposite side of the Petri dish, as illustrated in Figure S1. Five microliters of pure terpenes were poured on the paper, the Petri dishes were closed with parafilm and conserved at 25 °C. Selected terpenes (α-pinene, camphene, caryophyllene and humulene) were purchased from Sigma-Aldrich (L’lsle-d’Abeau, France). Terpene concentrations were chosen so that they were of the same order of magnitude as the natural terpene concentrations in carrot leaves. Indeed, terpene concentrations used in vitro were around 1 mM. Individual terpene concentrations in leaves of the carrot genotypes used in this work reached 0.3 mM and estimated total terpene amounts exceeded 200 mg/kg (representing about 2 mM when calculated for monoterpenes). In addition, the sequestration of terpenes in glandular trichomes probably results in *Alternaria dauci* facing much higher local terpene concentrations when infecting carrot leaves.

Mycelial growth was monitored by using pictures taken with a Nikon D5600 camera with an AF-S Nikkor 50 mm 1:1.4, at 0, 3, 4, 5 and 6 days. *A. dauci* growth was evaluated by measuring mycelium surface using Fiji software [27]. Two independent experiments were performed, each with three replicates per treatment. *T*-tests were performed using Excel.

## 3. Results

### 3.1. Consistency of rQTL among Years

In 2016, we detected less rQTL (4 rQTL) than in 2011 (5 rQTL) and 2014 (7 rQTL), and the phenotypic variation explained by the rQTL (global R^2^) was lower in 2016 (24%) than in 2011 (43%) and 2014 (58%). Nevertheless, the four rQTL found in 2016 overlapped with those identified during the two previous years with some differences in the confidence interval of each rQTL (Table S1). The most important and consistent QTL through the years was observed on chromosome 6. The heritability of disease score during the three years was very high, around 71%.

### 3.2. mQTL-rQTL Co-Localization Analysis Reveals Candidate Terpenes for Resistance to ALB

**Figure 1:**
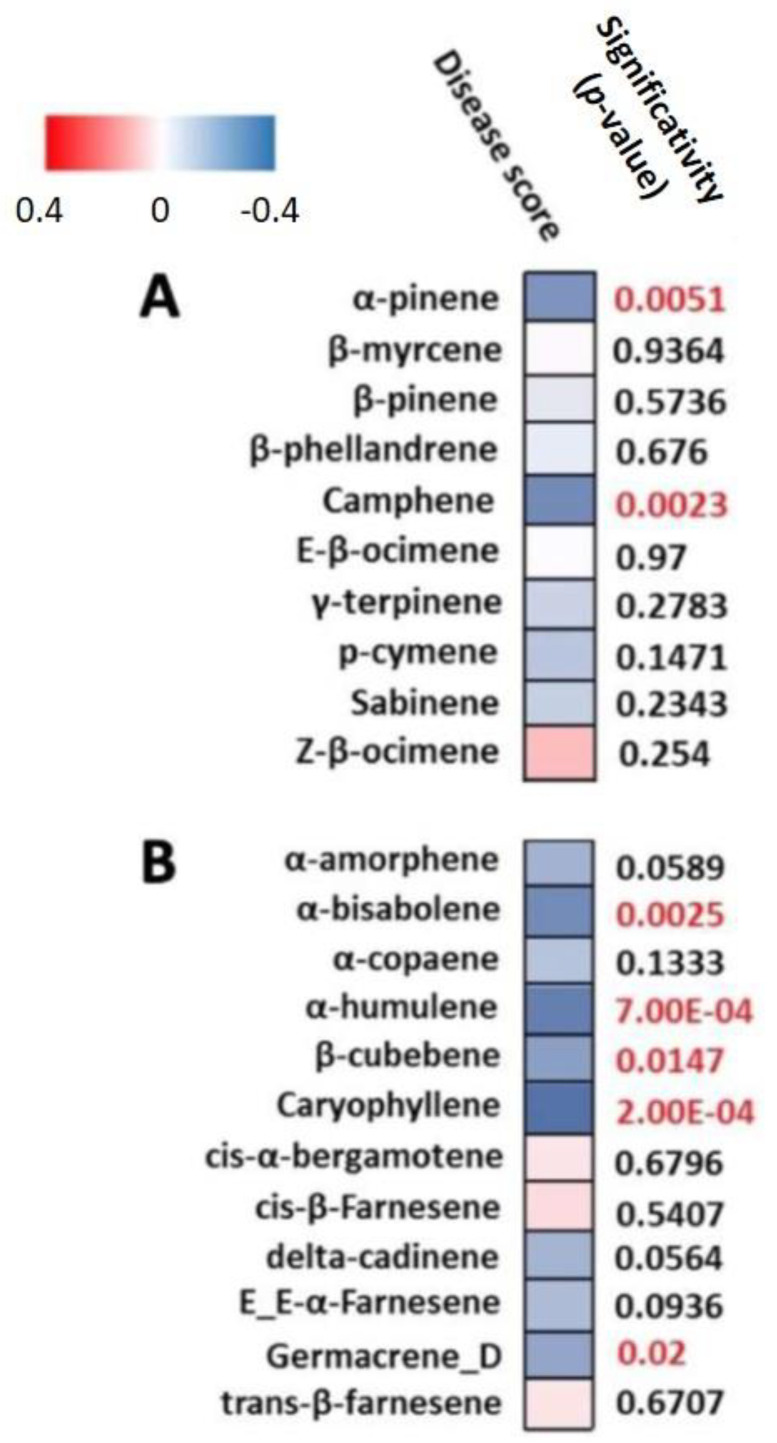
Spearman rank correlation between disease score and the contents of the 22 terpenes ((**A**) monoterpenes; (**B**) sesquiterpenes) that mQTL co-localized with rQTL. Red square indicates positive correlation and blue, negative correlation (i.e., the higher levels of these terpenes the lower the symptoms). Significance of correlation is given by the *p*-value. Red labels indicate *p*-values < 0.05.

Using terpene profiling data determined by HS-SPME-GC-MS, we performed an mQTL detection for 30 terpenes (15 monoterpenes and 15 sesquiterpenes, Table S2). We found mQTLs for 25 terpenes (11 monoterpenes and 14 sesquiterpenes). Among these 25 terpenes, 22 had mQTLs which co-localized with r-QTLs on chromosome (chr) 1, 2, 4, 6 and 8 with two hotspots on chr 4 and one hotspot on chr 8 (Figure S2). Furthermore, we performed a correlation analysis between terpene accumulation levels and ALB disease scores. Seven terpenes, namely α-pinene, camphene, α-bisabolene, α-humulene, β-cubebene, caryophyllene and germacrene D, showed significant negative correlation with disease score, i.e., higher levels of these terpenes were associated with lower symptoms (Figure 1).

**Figure 2:**
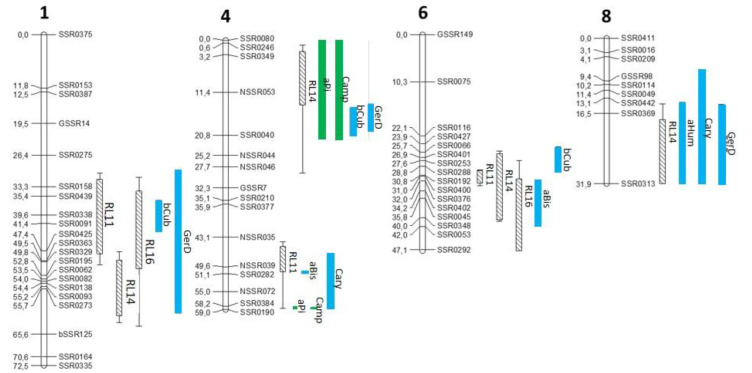
Co-localization between rQTL and mQTL of terpenes presenting significant negative correlation with ALB disease score. rQTL are named RL for resistance loci with the year of phenotyping. The confidence interval is bordered by two small vertical lines for the 1.5 LOD support interval (SI). A hatched area represents a 1 LOD SI. Metabolite-QTL is named with an abbreviation of each terpene: aPi = α-pinene, Camp = camphene, aBis = α-bisabolene, aHum = α-humulene, bCub = β-cubebene, Cary = caryophyllene, GerD = germacrene D. Monoterpene and sesquiterpene mQTL are represented with green and blue bars, respectively, with a 1 LOD SI.

mQTL associated with these seven terpenes co-localized with rQTL on chr 1, 4, 6 and 8 (Figure 2). For mQTL on chr 1, 4 and 6, favorable alleles for their accumulation and favorable alleles for resistance underlying the rQTL were brought by K3. For rQTLs on chr 8, H1 brought the favorable allele for resistance and the favorable alleles for terpene accumulation (α-humulene, caryophyllene and germacrene D). Metabolite accumulation explained by the mQTL (global R^2^) ranged between 12% (for α-humulene) and 58% (for α-pinene and camphene) (Table 1). The heritability varied from 31% (β-cubebene) to 65 and 68% for camphene and α-pinene, respectively. Camphene and α-pinene exhibited the same max position for their respective two mQTL on chr4 with approximately the same R^2^. These two metabolites shared the same max position with caryophyllene at the bottom of the chr 4. Germacrene D and β-cubebene also had the same max position on chr 1 and chr 4 (Figure 2 and Table 1).

### 3.3. Differential Expression Analysis of Genes Underlying the Co-Localization Regions

We compared gene expression in H1 and K3 lines 48 h after *A. dauci* inoculation. We gave special attention to genes under mQTL-rQTL co-localization areas. Candidate genes potentially related to terpene accumulation were selected based on their belonging to either the terpene synthase (TPS) or the transcription factors (TF) gene families. Such genes were identified at all co-localization sites and some of them were significantly differentially expressed between the resistant K3 and the susceptible H1 line (Table 2).

For chr 1, five genes coding for transcription factors (TF) were identified at the co-localization site, i.e., ethylene response factor (ERF), two basic leucine zipper (bZIP) and two WRKY. The genes coding for bZIPHY5 and the WRKY (WRKY33 and WRKY48) were overexpressed in K3 compared to H1, in contrast to those coding for ERF71 and bZIP53. However, no TPS gene was found at this site (Table 3).

Under the rQTL region RL14 at the top of chromosome 4, only two genes coding for transcription factors NAC29 and WRKY33 were significantly overexpressed in K3. In the same region, we also identified a gene encoding a germacrene D synthase-like enzyme (108217562).

Among the ten genes potentially related to terpenes at the co-localization site at the bottom of the same chromosome 4, we identified four TPS and six transcription factor genes. Three TPS genes were annotated as coding for terpene synthase 10-like [22] and correspond to DcTPS55, DcTPS26 and DcTPS54 using the most recent naming system proposed by Keilwagen et al. (2017). These genes were functionally characterized by [29,30]. The last TPS gene was annotated as β-bisabolene synthase-like gene. DcTPS55, DcTPS26 and DcTPS54, NAC2, ERF4 and bZIP17 were significantly overexpressed in K3, while the genes coding for β-bisabolene synthase-like gene, WRKY7, ERF054 and bZIP27, were underexpressed in K3 compared to H1 (Table 2 and Table 3). In addition, we identified a germacrene D synthase-like gene (108216912) and a TPS 10-like gene (108217598, DcTPS04), but they were not differentially expressed between resistant (K3) and susceptible (H1) genotypes.

In the co-localization region on chr 6 (Table 2), differentially expressed TF genes were bZIP61 and ERF4 with higher expression in K3 and SQUAMOSA-promoter binding protein 1 (SPL1) higher in H1 (Table 3). Finally, on chr 8, differentially expressed candidates were an α-farnesene synthase-like gene (Table 3) overexpressed in K3 and four genes coding for transcription factors with AP2/ERF and ERF1B-like higher in K3, while one MYB and ERF-like genes were underexpressed in K3 (Table 3).

### 3.4. Further Analysis of TPS Genes Overexpressed in K3

**Figure 3:**
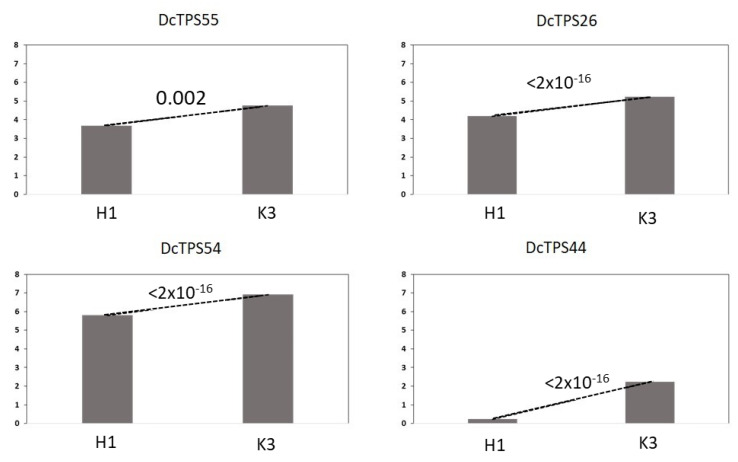
Relative expression of TPS genes located in the mQTL-rQTL co-localization regions on chromosomes 4 and 8 in H1 (susceptible) and K3 (resistant) genotypes. The number above the dotted line indicates significance of the difference (*p*-value).

Among the genes overexpressed in K3 in comparison to H1 (Figure 3), we identified three genes encoding monoterpene synthases DcTPS55, DcTPS26 and DcTPS54 genes under rQTL on chr 4 between NSSR039 and SSR0282. The four terpenes whose mQTL co-localized with the rQTL in this region were α-bisabolene, α-pinene, camphene and caryophyllene (Figure 2 and Table 1). Under rQTL on chr 8, we detected an α-farnesene synthase-like DcTPS44 gene overexpressed for K3 (Figure 3). It was consistent with the mQTL detected for α-farnesene on this chr 8 (Figure S2) but in our study, α-farnesene level was not correlated with resistance (Figure 1).

### 3.5. In Vitro Bioactivity of α-Pinene, Camphene, Caryophyllene and Humulene towards A. dauci

**Figure 4:**
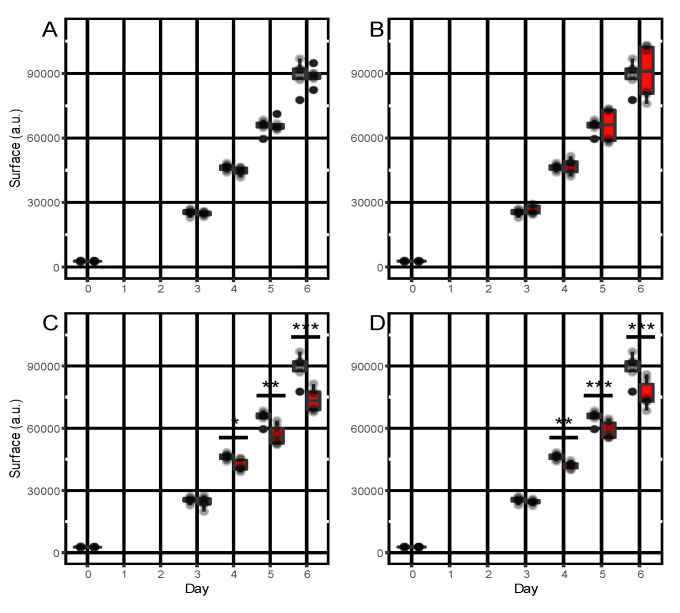
Inhibitory effect of terpenes on Alternaria dauci growth. Growth of Alternaria dauci was monitored as described in Section 2 (Materials and Methods). Two independent experiments with 3 replicates were performed. The surface of Alternaria mycelium was measured at days 0, 3 and 6. Controls are in gray and terpene assays in red. Tested terpenes were: (**A**) camphene, (**B**) α-pinene, (**C**) caryophyllene, (**D**) α-humulene. The symbols *, ** and *** indicate significantly different mycelium surfaces compared to control conditions using a T test, with *p*-value <0.05, <0.01 and <0.001, respectively.

The effect on fungal growth of four commercially available compounds among the seven candidate terpenes was assessed using an in vitro assay. By comparing mycelium growth in control conditions and in the presence of the selected terpenes, significant growth inhibition was observed with α-humulene and caryophyllene from the fourth day of the experiment. Growth inhibition persisted until the sixth day. Conversely, no significant growth inhibition was observed in these conditions with camphene and α-pinene (Figure 4).

## 4. Discussion

Quantitative trait loci detection is a good way to understand the genetic basis of variation associated with a quantitative trait [31]. Unfortunately, most of the time, the underlying mechanisms are poorly understood. In 2018, Koutouan et al. [17] identified fifteen terpenes which were more highly accumulated in the resistant genotype K3 than in the susceptible H1. The present study aimed at the identification of mQTL of terpenes co-localizing with rQTL in a segregating population obtained between H1 and K3. This mQTL-rQTL co-localization analysis was performed without a priori on all terpenes detected in carrot leaves, with no particular focus on the fifteen terpenes identified by [17].

This mQTL-rQTL co-localization approach identified camphene, α-pinene, α-bisabolene, α-humulene, β-cubebene, caryophyllene and germacrene D as candidate terpenes potentially associated with the resistance to *A. dauci*. Interestingly, these metabolites were previously identified as differentially accumulated between several resistant and susceptible genotypes by [17] except β-cubebene, and moreover they showed that camphene, α-pinene, α-bisabolene, α-humulene and caryophyllene were more accumulated in K3 compared to H1. For these five terpenes, we mapped mQTL but also β-cubebene and germacrene D, which were not differentially accumulated between parent H1 and K3. This last result was unexpected, as it seems more difficult to map QTL whatever the trait when there is no difference between parental lines for this trait. Keurentjes et al. [32] also identified mQTL for metabolites which were not differentially accumulated between parents. These detections could be explained by genome recombination between the two parental alleles. This result confirms that relying only on differential metabolite accumulation between parental lines is not sufficient to postulate the involvement of some metabolites in resistance, as the link between metabolite amounts and resistance may be fortuitous. For example, in the present study, only 7 of the 15 terpenes highlighted in the previous study by [17] were confirmed, while some new candidates have been identified, such as β-cubebene and germacrene D.

Through the mQTL-rQTL co-localization approach and a correlation test, we could associate seven metabolites with resistance. These seven terpenes were negatively correlated with disease score, which means that their accumulation is favorable for resistance. In addition, for a given chromosome, the parental origin of the favorable alleles (leading to metabolite accumulation) of mQTL was also the one bringing the favorable allele for resistance. Working on resistance of *Arabidopsis* to *Botrytis cinerea*, Rowe and Kliebenstein [33] used the same approach and highlighted that mQTL of camalexin co-localized with rQTL. They also indicated that both favorable alleles for camalexin accumulation and for resistance to *B. cinerea* were brought by the same genotype, which makes sense with the involvement of camalexin in resistance. On the same topic, Zhang et al. [34] showed co-localization between mQTLs of indole glucosinolates and rQTLs involved in *Arabidopsis* resistance to *B. cinerea*. They also showed that favorable alleles for resistance and indole glucosinolate accumulation came from the same resistant genotype.

In our analysis, mQTL for different terpenes often co-localized on the genome with the same maximal position and sometimes with the same R^2^. This could be explained by the fact that a single terpene synthase may catalyze the synthesis of different products. For example, Gambliel and Croteau [35] and Huber et al. [36] showed that the activity of pinene synthase (EC:4.2.3.119) could lead to an equal amount of α-pinene, camphene or β-pinene. Using the carrot genome published by [22], we searched for genes potentially involved in terpene synthesis within the co-localizing loci. Among these genes, our microarray analysis revealed differential gene expression for four TPS genes under mQTL-rQTL at the bottom of chromosome 4; three of them (DcTPS55, DcTPS26 and DcTPS54) were the ones identified by [30]. It is well known that in many plant genomes, terpene synthase genes are organized in clusters [37,38]. Working on carrot terpenes, Keilwagen et al. [28] identified in the same area a cluster of TPS on chr 4 with five terpene synthase genes. In the present study, two other TPS genes were identified in this same area, but they were not differentially expressed between H1 and K3.

Recently, Reichardt et al. [30] investigating this region of carrot genome identified a QTL cluster for sabinene, α-terpinene, γ-terpinene and terpinen-4-ol in leaves while in vitro assay identified DcTPS54 as a single product sabinene synthase. Additionally, among the 19 TPS biochemically characterized by Muchlinski et al. [29], recombinant DcTPS55, DcTPS26 and DcTPS54 proteins produced in vitro similar volatiles profiles such as sabinene, limonene, β-myrcene, α-pinene and α-terpineol from geranyl diphosphate, while DcTPS26 also produced several sesquiterpenes including β-bisabolene. At this time, there is no evidence that one of these TPS could be responsible for the accumulation of the mapped metabolite identified in this chr 4 region, but it is very likely. On chr 8, we also identified a gene coding for an α-farnesene synthase-like that showed similarity with known terpene synthases genes coding for germacrene D, caryophyllene and α-humulene. However, additional characterization of these TPS genes is necessary to investigate its involvement in the accumulation of these three co-localizing metabolites.

In addition to the TPS 10-like and α-farnesene-like genes, we also identified transcription factor genes that can be involved in the regulation of terpene accumulation. The role of the WRKY transcription factor in regulation of specialized metabolite is well known [39]. Tholl [3] was the first to review the involvement of the WRKY transcription factor in TPS regulation and the presence of W-box, the binding site of WRKY over numerous TPS. For example, Spyropoulou et al. [40] showed that WRKY73 could activate three TPS promoters from *Solanum lycopersicum*. Herein, we identified three different WRKY genes, two on chr 1 (WRKY33 and WRKY48) where germacrene D and β-cubebene mQTL mapped, and one WRKY33 on the top part of chr 4 where α-pinene, camphene, germacrene D and β-cubebene mQTL mapped. These three WRKY genes were overexpressed in K3, which coincided with favorable alleles for accumulation on co-localizing mQTL (α-pinene, camphene, germacrene D and β-cubebene). As far as we know, no WRKY33 or 48 have been identified in regulation of TPS, but their role in resistance to pathogens has been highlighted, especially for WRKY33. Zheng et al. [41] indicated that WRKY33 was involved in *Arabidopsis* resistance to *Botrytis cinerea* and *Alternaria brassicicola* through activation of the jasmonate biosynthesis pathway, which itself regulated activation of defense genes. In a previous work, Rodriguez et al. [42] demonstrated that downregulation of limonene synthase in orange peel enhanced resistance to *Penicillium digitatum*. Afterward, they showed that orange line AS7 (antisense 7), which was downregulated for limonene synthase gene, overexpressed a putative gene encoding the WRKY33 [43]. In our case, the WRKY33 could be part of a signal cascade to induce resistance by triggering TPS synthesis.

In addition to WRKY, we also identified other transcription factor families. The bZIP TFs were found on almost all the co-localization sites except on chr 8. Among all, the HY5 overexpressed by K3 has been shown by Zhou et al. [44] to be a positive regulator of β-pinene synthase gene in *Artemisia annua* L. Two NAC domain transcription factors (TF), under chr 4, were overexpressed by K3. These NAC transcription factors were NAC2 and NAC29. Interestingly, the NAC2 was shown to bind to the promoter of a kiwi (*Actinidia arguta*) *Aa*TPS1 gene, which resulted in an overexpression of the gene, overaccumulation of the protein and a high level of terpinolene, myrcene, limonene, α-pinene and linalool [45]. Ethylene response factor (ERF) TF was found under all of our mQTL-rQTL co-localization areas except for the top of chr 4. Their overexpression was divergent, ERF4 on chr 4 and chr 6 (108217832 and 108225207) and ERF1B-like (108198802) were overexpressed in K3 at the opposite of the three others on chr 1, 4b and 8. One of these ERF, ERF71 has been identified by Li et al. [46] as upregulator (activator) of geraniol synthase, which catalyzes the synthesis of the monoterpene E-geraniol in *Citrus sinensis*. In our pathosystem, if ever ERF71 would also catalyze synthesis of monoterpens, it could result in fewer quantities of sesquiterpens. This could be consistent with the unfavorable alleles for accumulation of β-cubebene and germacrene D observed for H1 on chr 1. Clearly, further analyses are needed to decipher ERF impacts within carrot *A. dauci* interaction. Finally, the last family of TF identified was the SQUAMOSA binding protein 1-like (SPL1) found on chr 6 and overexpressed by H1. Yu et al. [47] indicated that an SPL TF was activator of a TPS21 that catalyzes the biosynthesis of caryophyllene in *Arabidopsis*. As previously suggested for ERF71, if ever this SPL TF would also play a role in the accumulation of the α-bisabolene and β-cubebene in carrot, which mQTL co-localized with this TF gene, we hypothesized SPL TF would be a downregulator of this accumulation because favorable allele for accumulation of mQTL of α-bisabolene and β-cubebene came from K3.

Based on the co-localization between metabolic and resistance QTL, we proposed that some terpenes could be part of the resistance mechanism against the fungus *A. dauci*. In order to substantiate this hypothesis, we tested the potential fungitoxic properties of four of these terpenes, which were commercially available. Indeed, significant mycelial growth inhibition was observed in the presence of α-humulene and caryophyllene. Conversely, camphene and α-pinene did not show any significant effect on fungal growth. However, relative quantification of these two last compounds in leaf samples showed three times higher concentration in the resistant K3 line than in the susceptible H1 [17] suggesting together with mQTL/rQTL co-localization that they could play a role in the resistance of carrot to *A. dauci*. These apparently contradictory results suggest that depending on the metabolite, different stages of the life cycle of the fungus could be affected, which makes sense when considering the mQTL/rQTL co-localization with the four terpenes: while the two monoterpene α-pinene and camphene mQTL co-localized with rQTL on chromosome 4, the two sesquiterpene α-humulene and caryophyllene mQTL were mainly co-localizing with rQTL on chromosome 8. We previously concluded that these different rQTLs could act on different components of quantitative resistance, some of them being able to reduce the efficiency of fungal penetration while others delay the invasion and growth of the pathogen in the leaf [16]. In this hypothesis, α-humulene and caryophyllene may impact fungal growth, as determined by our assay, while α-pinene and camphene may act on a different, but not yet clearly identified fungal target. Preliminary results suggested that it could be around conidia germination, as illustrated in Figures S3 and S4.

Literature already reported the involvement of some of these terpenes in resistance to some pathogens using in vitro assay. Hammer et al. [48] showed fungistatic and fungicidal activity of α-pinene against *Aspergillus niger, A. flavus* and *A. fumigatus*. Sati et al. [49] demonstrated that essential oil of *Artemisia nilagirica*, in which germacrene D, caryophyllene and camphene are main constituents, had significant antifungal effect against the plant pathogenes *Rhizoctonia solani*, *Sclerotium rolfsii* and *Macrophomina phaseolina*. Caryophyllene alone inhibited mycelial development of *Aspergillus niger,* which may be a pathogen of both plants and humans [50]. García-Rellán et al. [51] showed that essential oil from *Satureja cuneifolia*, containing camphene and camphor, exhibited strong antifungal activity against *Phytophthora palmivora* and *Phaemoniella chlamydospora*. Interestingly, the reported antifungal activity of terpenes was mostly shown through essential oils with different components, suggesting that the seven terpenes identified here could act in a synergistic way for carrot resistance to *A. dauci*.

The roles of metabolite in plant resistance to pathogens have been suggested across an abundance of literature; however, few studies have combined genetic and metabolomic approaches to investigate metabolite-associated defenses [52]. Herein, through an mQTL-rQTL co-localization approach, we highlighted seven terpenes that were strongly associated with carrot resistance to *A. dauci.* Using transcriptomics and genome analysis, we identified potential enzymes and regulators involved in the biosynthesis of these terpenes. Finally, functional analyses revealed that two of these terpene candidates, α-humulene and caryophyllene, exhibited fungitoxic properties, consistent with a direct role of these compounds in disease resistance. Future work will be aimed at evaluating the role of other candidate metabolites or combinations of them on carrot resistance to *A. dauci*.

## Figures and Tables

**Table 1:** Characteristics of terpene mQTL co-localizing with rQTL and significantly correlated with disease score on carrot leaves after *A. dauci* attack. SI = support interval; Chr = chromosome; RL= resistance loci; R^2^ = explained phenotypic variation; cM = centimorgan. aPi = α-pinene, Camp = camphene, aBis = α-bisabolene, aHum = α-humulene, bCub = β-cubebene, Cary = caryophyllene, GerD = germacrene D; ^t^ and ^b^ = top and bottom of Chr respectively.

Code of mQTL	Chr	1-LOD SI (cM)	Max Position (cM)	R^2^ (%)	Global R^2^ (%)	Heritability *H*^2^ (%)	Additive Effect of Allele	
							H1	K3
aPi	4 ^t^	0–21.6	20.4	11.4	58.3	68	−0.157	0.157
	4 ^b^	58.3–58.7	58.7	42.9			−0.388	0.388
Camp	4 ^t^	0–21.7	20.4	10.7	57.6	65	−0.153	0.153
	4 ^b^	58.4–58.7	58.7	42.5			−0.389	0.389
aBis	4 ^b^	50.6–51	50.8	51.7	53.1	34	−0.436	0.436
	6	31.8–41.9	40.2	9.7			−0.140	0.140
aHum	8	14.1–31.9	16.4	12.2	12.2	39	0.209	−0.209
bCub	1	36.3–43.2	39.6	20.5	41.7	31	−0.263	0.263
	4 ^t^	14.7–20.8	16.4	26.9			−0.273	0.273
	6	24.6–30.1	28.9	12.6			−0.167	0.167
Cary	4 ^b^	46.6–58.7	58.7	9.3	18.6	33.3	−0.161	0.161
	8	6.9–31.9	11.3	9.1			0.174	−0.174
GerD	1	29.7–61	39.6	10	46	36.2	−0.173	0.173
	4 ^t^	13.9–19.8	16.4	26.4			−0.271	0.271
	8	14.5–31.9	21.4	18.7			0.235	−0.235

**Table 2:** Number of all genes and genes differentially expressed (GDE) identified by microarray analysis under mQTL-rQTL co-localization with special emphasis to the GDE potentially related to terpenes. ^t^ and ^b^ = top and bottom of chromosome (Chr) respectively.

Chr	Located Genes	Genes Differentially Expressed between K3 and H1		
		Underexpressed	Overexpressed	Related to Terpenes
1	2181	207	206	5
4 ^t^	1229	76	92	2
4 ^b^	1718	154	196	10
6	577	63	45	3
8	1025	100	105	5

**Table 3:** Terpene synthase and transcription factor genes underlying co-localization regions, which are differentially expressed between H1 (susceptible) and K3 (resistant) genotypes. TF = transcription factor; TPS = terpene synthase; ^t^ and ^b^ = top and bottom of chromosome (Chr), respectively. Dc refers to TPS naming system proposed by [28].

Chr	Gene Name (Locus Number)	Function	Relative Expression		*p*-Value
			H1	K3	
1	bZIPHY5 (108204232)	TF	3.94	4.48	0.004
	WRKY33 (108204915)	TF	2.65	4.64	<2 × 10^−16^
	WRKY48 (108204668)	TF	2.67	3.79	<2 × 10^−16^
	ERF71 (108206243)	TF	0.98	0.15	0.0004
	bZIP53 (108206338)	TF	3.28	2.23	<2 × 10^−16^
4 ^t^	NAC29 (108218926)	TF	4.44	5.90	0.0004
	WRKY33 (108219317)	TF	4.00	6.21	<2 × 10^−16^
4 ^b^	Terpene synthase 10-like (DcTPS55;108217470)	TPS	3.68	4.76	0.002
	Terpene synthase 10-like (DcTPS26;108217599)	TPS	4.19	5.22	<2 × 10^−16^
	Terpene synthase 10-like (DcTPS54;108217617)	TPS	5.80	6.92	<2 × 10^−16^
	β-bisabolene synthase-like (108216085)	TPS	6.67	5.67	0.0004
	WRKY7 (108215789)	TF	5.39	4.74	0.0008
	ERF054 (108216387)	TF	3.45	2.1	<2 × 10^−16^
	NAC2 (108215781)	TF	4.51	5.26	0.0004
	ERF4 (108217832)	TF	1.87	2.87	<2 × 10^−16^
	bZIP17 (108218833)	TF	3.98	4.76	0.0004
	bZIP27 (108217633)	TF	3.60	0.51	<2 × 10^−16^
6	bZIP61 (108225065)	TF	4.28	4.89	0.007
	ERF4 (108225207)	TF	0.82	3.29	0.002
	SPL1 (108224238)	TF	3.98	2.84	<2 × 10^−16^
8	α-farnesene synthase-like(DcTPS44; 108198720)	TPS	0.23	2.23	<2 × 10^−16^
	AP2/ERF (108198780)	TF	0.92	5.22	<2 × 10^−16^
	ERF1B-like (108198802)	TF	1.35	3.21	<2 × 10^−16^
	MYB (108197621)	TF	0.74	0.19	0.005
	ERF_like (108197006)	TF	4.38	2.66	<2 × 10^−16^

## Data Availability

The data presented in this study are available in the article and Supplementary Materials.

## Supplementary Materials

The following supporting information can be downloaded at: https://www.mdpi.com/article/10.3390/metabo13010071/s1, Figure S1: Illustration of the fungal growth inhibition assay; Figure S2: Co-localization of 22 terpene mQTL with rQTL; Figure S3: Evaluation of the inhibitory effect of α-pinene and camphene (7.34 mM) on germination of *Alternaria dauci* P2 strain after 4 and 70 h of incubation; Figure S4: Evaluation of the inhibitory effect of α-pinene and camphene on growth of *Alternaria dauci* P2 strain after 8 days of incubation; Table S1: List of quantitative resistance loci (rQTL) detected by connected analysis; Table S2: List of all terpenes per family.
