# Supplementary material for: Co-Localization of Resistance and Metabolic Quantitative Trait Loci on Carrot Genome Reveals Fungitoxic Terpenes and Related Candidate Genes Associated with the Resistance to Alternaria dauci
Source: Metabolites. 2023 Jan 2;13(1):71. doi: 10.3390/metabo13010071 (PMC9863879; doi:10.3390/metabo13010071)
Supplement: Supplementary file 1 [file metabolites-13-00071-s001.zip › Figure S1.pdf]

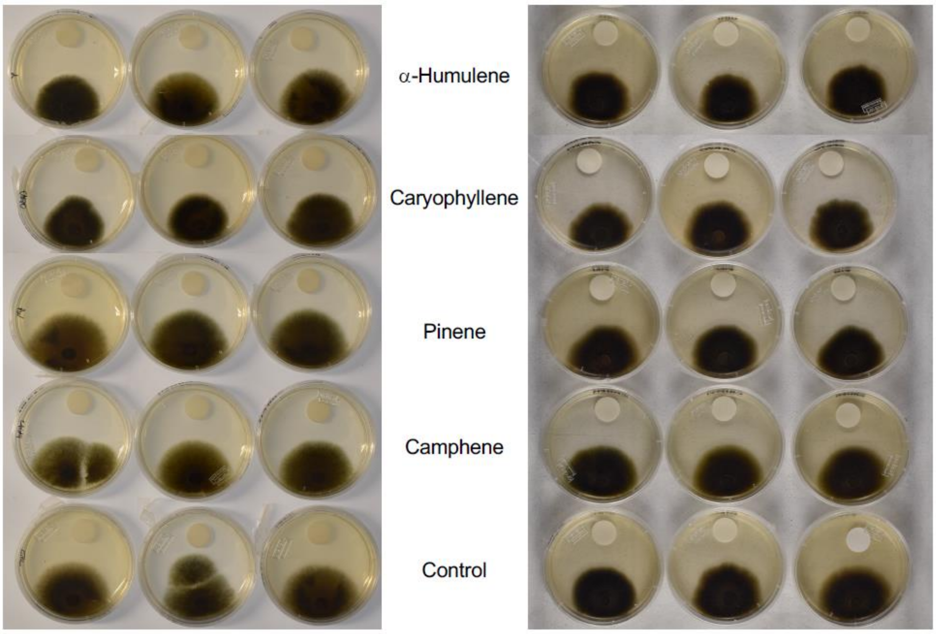

Figure S1. Fungal growth inhibition assay, 6 days post inoculation.

The *Alternaria dauci* P2 (FRA017) strain was inoculated with a 5 mm diameter punch, which was deposited on the side of a 5 cm diameter malt/agar Petri dish. Tested terpenes were deposited on a sterile antibiotic assay paper at the opposite side of the Petri dish. Petri dishes were closed with parafilm and conserved at 25°C. Two independent experiments with 3 replicates each were performed. Surface of Alternaria mycelium was measured at days 0, 3 to 6 as described in the Material and Method. Pictures represent the fungal growth 6 days post inoculation in control conditions and in the presence of the indicated terpenes.
