# Supplementary material for: Co-Localization of Resistance and Metabolic Quantitative Trait Loci on Carrot Genome Reveals Fungitoxic Terpenes and Related Candidate Genes Associated with the Resistance to Alternaria dauci
Source: Metabolites. 2023 Jan 2;13(1):71. doi: 10.3390/metabo13010071 (PMC9863879; doi:10.3390/metabo13010071)
Supplement: Supplementary file 1 [file metabolites-13-00071-s001.zip › Figure S3.pdf]

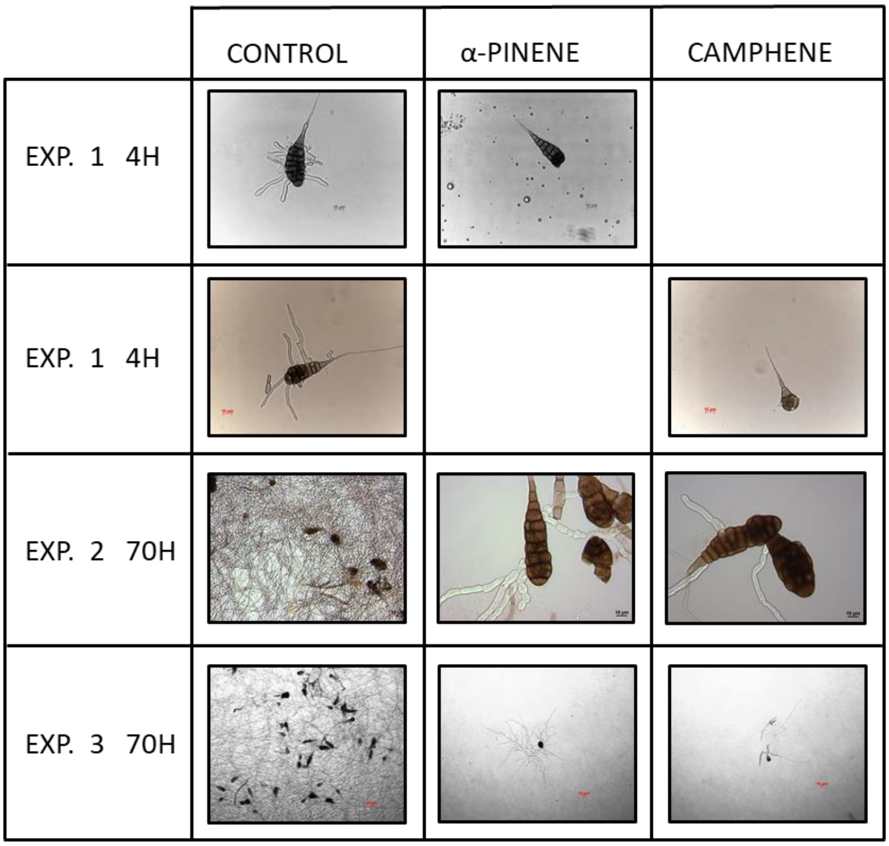

Figure S3. Evaluation of the inhibitory effect of α-pinene and camphene (7.34mM) on germination of *Alternaria dauci* P2 strain after 4 and 70 hours of incubation. Bar = 10 µm
