# Supplementary material for: Co-Localization of Resistance and Metabolic Quantitative Trait Loci on Carrot Genome Reveals Fungitoxic Terpenes and Related Candidate Genes Associated with the Resistance to Alternaria dauci
Source: Metabolites. 2023 Jan 2;13(1):71. doi: 10.3390/metabo13010071 (PMC9863879; doi:10.3390/metabo13010071)
Supplement: Supplementary file 1 [file metabolites-13-00071-s001.zip › Figure S4.pdf]

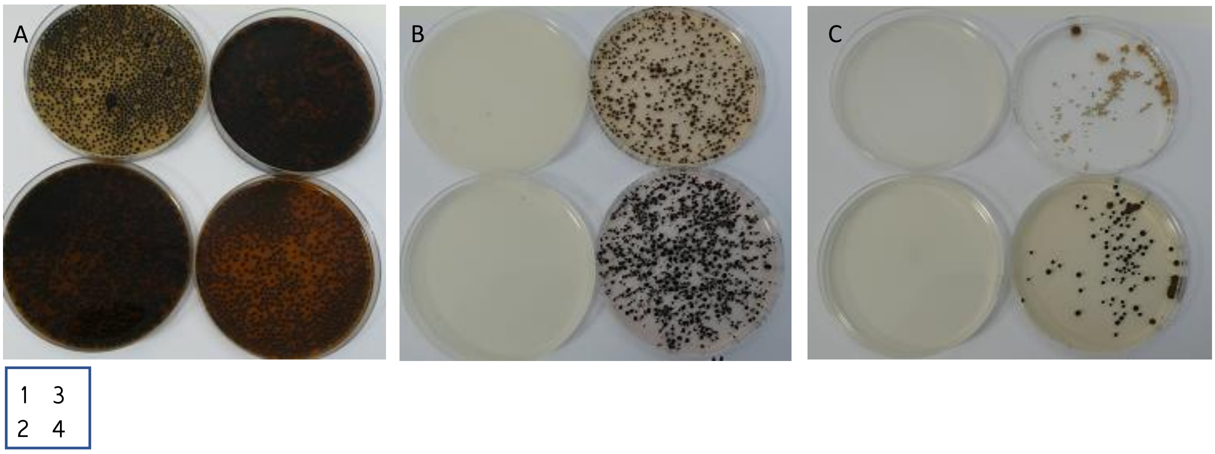

Figure S4. Evaluation of the inhibitory effect of α-pinene and camphene on growth of *Alternaria dauci* P2 strain after 8 days of incubation. Four concentrations of each terpene were tested 1: 14.6mM, 2: 7.34mM, 3: 1.47mM, 4: 0.73mM. A: control; B: α- pinene, C: camphene
