# Supplementary material for: Co-Localization of Resistance and Metabolic Quantitative Trait Loci on Carrot Genome Reveals Fungitoxic Terpenes and Related Candidate Genes Associated with the Resistance to Alternaria dauci
Source: Metabolites. 2023 Jan 2;13(1):71. doi: 10.3390/metabo13010071 (PMC9863879; doi:10.3390/metabo13010071)
Supplement: Supplementary file 1 [file metabolites-13-00071-s001.zip › Table S1.pdf]

Table S1. List of quantitative resistance loci (rQTL) detected by connected analysis. (SI = Support Interval; Chr = Chromosome; RL= Resistance Loci; R² = Explained phenotypic variation; cM = centimorgan)

Scoring year	Code of rQTL	Chr	1.5-LOD SI (cM)	1-LOD SI (cM)	Max position (cM)	R² (%)	Global R² (%)	Additive effect of allele		
								H1	I2	K3
2011	RL11	1	30.4 – 50.4	31.7 – 48	39.6	7	43	0.205	0.053	-0.259
		2	9.6 – 21.4	7.7 – 23.7	13.7	8		-0.203	0.204	-0.001
		3	0 – 15.1	0 – 12.7	5	12.4		0.267	-0.374	0.107
		4	44.1 – 58.7	45.2 – 50.7	47.7	13.9		0.106	0.347	-0.453
		6	29.6 – 33.1	29.7 – 32.5	32.1	21.6		0.336	-0.017	-0.318
2014	RL14	1	47.6 - 63.1	49.4 – 61.6	54.4	11.7	58	0.249	-0.171	-0.078
		2	8.4 - 35.6	9.7 - 22.3	13.7	13.4		-0.277	0.249	0.027
		3	0 - 15.9	0.5 - 15	10	12.5		0.262	-0.450	0.187
		3	41.7 -50.9	42.4 - 49.4	43.8	6.5		0.203	-0.046	-0.157
		4	1.1 - 29.1	2.5 - 14.2	8.3	11.2		0.0983	0.287	-0.385
		5	52.3 – 72	55.2 – 72	68.3	10		-0.248	0.290	-0.043
		6	25.5 - 40.9	26 - 40.5	27.7	20.2		0.309	0.034	-0.343
		8	14.4 - 31.9	17.8 – 31.9	31.4	8.5		0.006	-0.336	0.329
2016	RL16	1	31.3 - 63.9	34.1 - 51.3	39.6	7.3	24	0.246	-0.278	0.031
		2	1.8 - 42.7	4.7 - 23.6	18.1	7		-0.182	0.479	-0.298
		3	12.6 - 47.6	19.1 - 46.3	43.8	7.4		0.245	-0.078	-0.167
		6	27.6 - 47.3	31.6 - 47.3	47.2	6		0.163	0.124	-0.287
