# Supplementary material for: Co-Localization of Resistance and Metabolic Quantitative Trait Loci on Carrot Genome Reveals Fungitoxic Terpenes and Related Candidate Genes Associated with the Resistance to Alternaria dauci
Source: Metabolites. 2023 Jan 2;13(1):71. doi: 10.3390/metabo13010071 (PMC9863879; doi:10.3390/metabo13010071)
Supplement: Supplementary file 1 [file metabolites-13-00071-s001.zip › Table S2.pdf]

Table S2. List of all terpenes per family

Code	Full name	Family	Sub-family
aPi	α-Pinene	Terpene	Monoterpene
Camp	Camphene	Terpene	Monoterpene
bPi	β-Pinene	Terpene	Monoterpene
Sab	Sabinene	Terpene	Monoterpene
bMyr	β-Myrcene	Terpene	Monoterpene
Lim	Limonene	Terpene	Monoterpene
bPhe	β-Phellandrene	Terpene	Monoterpene
ZbOc	(Z)-β-Ocimene	Terpene	Monoterpene
gTer	γ-Terpinene	Terpene	Monoterpene
EbOc	(E)-β-Ocimene	Terpene	Monoterpene
pCy	p-Cymene	Terpene	Monoterpene
aTer	α-Terpinolene	Terpene	Monoterpene
cRo	cis-Rose oxide	Terpene	Monoterpene
Lin	Linalol	Terpene	Monoterpene
Bac	Bornyl acetate	Terpene	Monoterpene
aCop	α-Copaene	Terpene	Sesquiterpene
bCub	β-Cubebene	Terpene	Sesquiterpene
caBer	cis-α-Bergamotene	Terpene	Sesquiterpene
taBer	trans-α-Bergamotene	Terpene	Sesquiterpene
Cary	Caryophyllene	Terpene	Sesquiterpene
cbFar	cis-β-Farnesene	Terpene	Sesquiterpene
aHum	α-Humulene	Terpene	Sesquiterpene
tbFar	trans-β-Farnesene	Terpene	Sesquiterpene
aAmo	α-Amorphene	Terpene	Sesquiterpene
GerD	Germacrene D	Terpene	Sesquiterpene
bSel	β-Selinene	Terpene	Sesquiterpene
bBis	β-Bisabolene	Terpene	Sesquiterpene
EaFar	(E-E)-α-Farnesene	Terpene	Sesquiterpene
dCad	δ-Cadinene	Terpene	Sesquiterpene
aBis	α-Bisabolene	Terpene	Sesquiterpene
