# Supplementary material for: Co-Localization of Resistance and Metabolic Quantitative Trait Loci on Carrot Genome Reveals Fungitoxic Terpenes and Related Candidate Genes Associated with the Resistance to Alternaria dauci
Source: Metabolites. 2023 Jan 2;13(1):71. doi: 10.3390/metabo13010071 (PMC9863879; doi:10.3390/metabo13010071)
Supplement: Supplementary file 1 [file Figure S2.pdf › Figure S2.pdf]

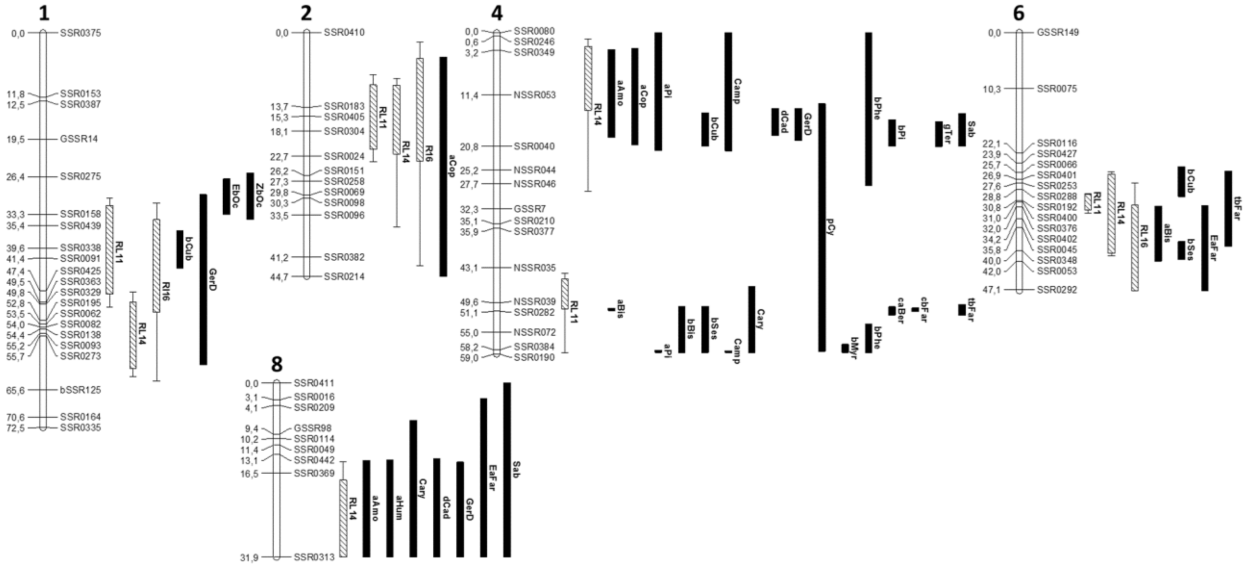

Figure S2. Co-localization of 22 terpene mQTL with rQTL. rQTL are named RL for resistance loci with the year of phenotyping. The confidence interval is bordered by two small vertical lines for the 1.5 LOD support interval (SI). A hatched area represents a 1 LOD SI. Metabolite-QTL are named with an abbreviation of each terpene as described in supplemental table 2.
